# Nomogram for predicting overall and cancer-specific survival in patients with postoperative follicular thyroid cancer

**DOI:** 10.1016/j.bjorl.2025.101719

**Published:** 2025-09-25

**Authors:** Xin Liu, Suidan Chen, Cangui Wu

**Affiliations:** a The First Affiliated Hospital of Wenzhou Medical University, Department of Pathology, Wenzhou, China; b Jieyang People’s Hospital, Department of Breast Oncology, Jieyang, Guangdong, China

**Keywords:** Follicular thyroid cancer, Nomogram, SEER, Prognosis, Overall survival

## Abstract

**Objectives:**

In this research, nomograms were constructed from clinical variables in patients with postoperative Follicular Thyroid Cancer (FTC) to predict the survival of FTC patients.

**Methods:**

Adult patients surgically treated for FTC from 2004 to 2015 were selected from the Surveillance, Epidemiology, and End Results (SEER) database. Significant clinical variables were screened using univariate analysis and further screened with multivariate Cox regression analysis, which were used to construct nomogram models for Overall Survival (OS) and Cancer-Specific Survival (CSS).

**Results:**

Age, sex, marital status, tumor size, glandular invasion status, N stage and M stage were identified as the clinical variables affecting the OS in FTC patients. Additionally, all variables, except sex, were found to have an impact on the CSS in FTC patients. The c-index shows agreement between the predicted results and the actual observed results, and the calibration curve of the probability of survival also shows agreement between the predicted plot and the actual observations.

**Conclusions:**

Our study successfully built a nomogram of OS and CSS for FTC patients who had undergone surgery.

**Level of evidence:**

IV: Retrospective cohort study.

## Highlights

• Determine the influencing factors that affect the prognosis of patients with FTC.
• Prediction of postoperative survival of FTC patients by Nomogram.
• Helps to assess disease prognosis and select treatment options.

## Introduction

According to the 2022 U.S. Cancer Report, the number of new cancer cases in the endocrine system is projected to be 47,050; of which are 43,800 cases of thyroid cancer accounting for approximately 93% of all endocrine system tumors.^1^ Thyroid tumors are classified as benign, low risk, or malignant. According to the 2022 World Health Organization (WHO) classification of thyroid tumors, thymic, salivary gland, and germ cell tumors may present as primary thyroid tumors.^2^ PTC is the most common form of thyroid cancer, consisting of papillary structures, and its pathologic diagnosis relies solely on the typical nuclear cytologic manifestations.^3^ PTC is a relatively mild cancer with a good prognosis, and surgery is often straightforward. However, thyroidectomy decisions for thyroid microcarcinoma patients should be made more cautiously to prevent post-operative decision regret.^4^^,^^5^ FTC is the second most common thyroid cancer after PTC and is characterized by follicular formation and lacks the nuclear features of PTC. Unlike PTC, which often appears in the form of multicenter tumors, FTC usually manifests as a single tumor.^6^ Compared with PTC, FTC tends to be more aggressive, with the potential for local invasion and distal metastasis to the bone and lungs, with a poorer average prognosis and greater cancer-specific mortality, and surgical resection is the main option for the treatment of FTC.^7^ Hürthle Cell Carcinoma (HCC) is one of the histologic classifications of thyroid cancer, and the 2022 WHO classification has replaced “Hürthle cell” with “carcinoma”. This change has led to Hürthle cell adenomas and HCC being reclassified as Oncocytic Adenomas and Oncocarcinomas (OCAs).^2^ HCC is no longer a subtype of FTC but is now a separate subtype of thyroid cancer that accounts for approximately 5% of all are well-differentiated but has the highest risk of metastasis.^8^ PTC, FTC and HCC also known as Differentiated Thyroid Cancer (DTC) are the most common subtypes of thyroid cancer, accounting for more than 90% of all thyroid cancers.^9^ Current research primarily focuses on PTC, while studies on FTC are relatively limited. Preoperative diagnosis of FTC remains a clinical challenge, and there is no consensus on its prognostic factors.^10^ In this research, we derived prognostic factors affecting FTC by evaluating clinical variables in postoperative FTC patients, and then constructed nomograms to predict the survival of FTC patients.

SEER is a public database that collects information about cancer from the U.S. population, and it provides data related to survival and death of cancer patients, and the data are expanding.^11^ Column line graphs play an integral role in cancer prognosis. It enables the estimation of individualized risk based on patient disease characteristics and can also incorporate continuous variables and relevant disease determinants into the prognosis to inform clinicians.^12^ In this research, meaningful clinical variables were used to construct nomograms so as to assess the survival of patients with FTC and to target progressive clinical prediction and treatment according to the patient's condition.

## Methods

### Patient data and methods

Age, sex, race, marital status, age at diagnosis, tumor size, glandular invasion, number of lesions, surgical modality, radiotherapy modality, TNM stage, and survival status variables were extracted from the SEER database. The following criteria were included: (1) Aged ≥18-years at diagnosis and had undergone surgery; (2) Had a diagnosis between 2004–2015; (3) Had the International Classification of Disease (ICD) code C73.9 with FTC pathology codes 8330/3, 8331/3, 8332/3, and 8335/3; and (4) Had a pathological diagnosis of FTC. The exclusion criteria were as follows: (1) Incomplete clinical data (age, survival time, tumor size, glandular invasion status, TNM staging, etc.; and (2) Cases were confirmed by autopsy or death report.

### Statistical analysis

We retrieved 8371 cases from the SEER database, excluded incomplete clinical information, and 5639 cases met the inclusion criteria. The data from 5639 cases were used to derive the best cut-off values for age and tumor size using X-tile software. Clinical variables of FTC patients were analyzed using univariate analysis, followed by multivariate Cox regression analysis to derive risk factors affecting the prognosis of FTC patients. The accuracy of nomogram in predicting the survival of FTC patients was evaluated using the subject Operating Characteristic Curves (ROCs) and calibration curves. The results were considered statistically significant at p < 0.05.

## Results

### Clinicopathologic characteristics

**Fig. 1:**
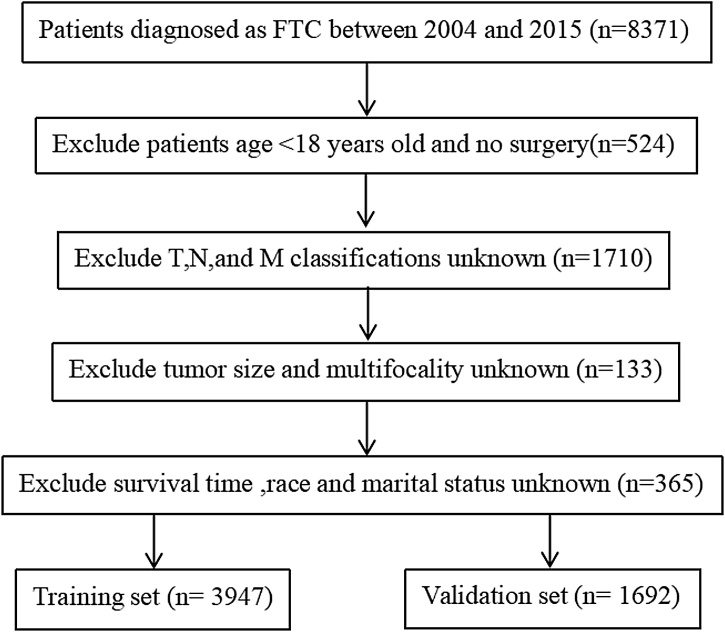
The screening process.

**Fig. 2:**
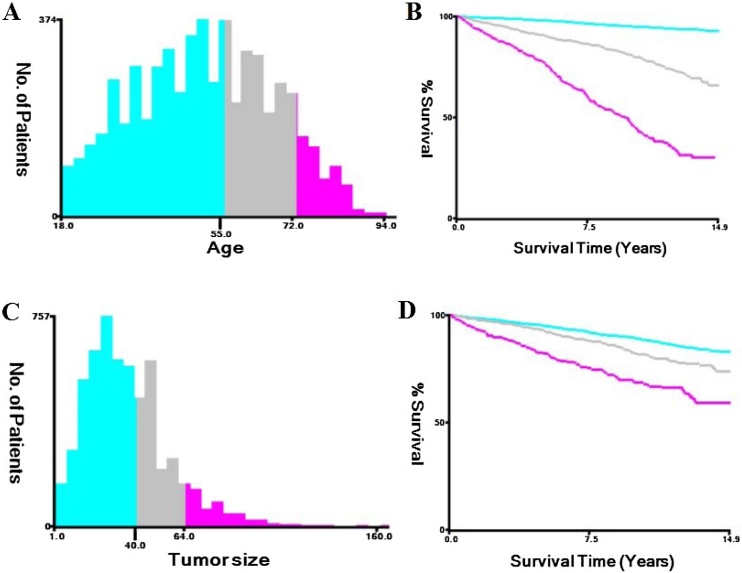
Two optimal cutoff values for age (A) and tumor size (C) were obtained by X-tile analysis of 5639 cases. Three categorical variables were used for age (B) and tumor size (D), and the survival curves of the three groups were clearly separated.

The 5639 patients who met the inclusion criteria were randomly grouped at a ratio of 7:3, with 3947 patients in the training set and 1692 patients in the validation set. The selection process is shown in Fig. 1. Age and tumor size were grouped into three categorical variables by X-tile software (Fig. 2). According to the clinicopathological characteristics of the patients, the majority of patients who underwent FTC were diagnosed with the following characteristics: aged <56-years in 59.1% of patients, female in 70.1% of patients, white ethnicity in 78.4% of patients, married in 61.9% of cases, tumor size <41 mm in 67.6% of cases, glandular ectasia in 91.5% of cases, lesion singularity in 86.2% of cases, complete thyroidectomy in 73.5%, radioisotope or radiation beam plus isotope or implant in 55.1%, T2 stage cancer in 40.9%, no lymph node metastasis in 97.6% and distant metastasis in 96.8% (Table 1).

**Table 1:** Demographic and pathological characteristics of postoperative FTC patients.

Variables	All patients (%) (n = 5639)	Training set (%) (n = 3947)	Validation set (%) (n = 1692)
Age			
<56	3331 (59.1)	2346 (59.4)	985 (58.2)
56–72	1688 (29.9)	1188 (30.1)	500 (29.6)
>72	620 (11.0)	413 (10.5)	207 (12.2)
Sex			
Male	1671 (29.6)	1164 (29.5)	507 (30.0)
Female	3968 (70.4)	2783 (70.5)	1185 (69.9)
Race			
White	4423 (78.4)	3057 (77.5)	1366 (80.7)
Black	674 (12.0)	481 (12.2)	193 (11.4)
Other	542 (9.6)	409 (10.4)	133 (7.9)
Marital status			
Married	3493 (61.9)	2446 (62.0)	1047 (61.9)
Unmarried	2146 (38.1)	1501 (38.0)	645 (38.1)
Tumor size			
<41	3813 (67.6)	2663 (67.5)	1150 (68.0)
41‒64	1262 (22.4)	898 (22.8)	364 (21.5)
>64	564 (10.0)	386 (9.8)	178 (10.5)
Extension			
No	5162 (91.5)	3612 (91.5)	1550(91.6)
Yes	477 (8.5)	355 (9.0)	142(8.4)
Extension			
No	5162 (91.5)	3612 (91.5)	1550 (91.6)
Yes	477 (8.5)	355 (9.0)	142 (8.4)
Multifocality			
Unifocal	4858 (86.2)	3389 (85.9)	1469 (86.8)
Multifocal	781 (13.8)	558 (14.1)	223 (13.2)
Surgery			
Lobectomy	1161 (20.6)	822 (20.8)	339(20.0)
Subtotal or near-total thyroidectomy	336 (6.0)	232 (5.9)	104(6.2)
Total thyroidectomy	4142 (73.5)	2893 (73.3)	1249(73.8)
Radiation			
None or refused	2381(42.2)	1671(42.3)	710(42.0)
Radiation Beam or Radioactive implants	149 (2.6)	106 (2.7)	43 (2.5)
Radioisotopes or Radiation beam plus isotopes or implants	3109 (55.1)	2170 (55.0)	939 (55.5)
T			
T1	1350 (23.9)	937 (23.7)	413 (24.4)
T2	2309 (40.9)	1621 (41.1)	688 (40.7)
T3	1858 (32.9)	1299 (32.9)	559 (33.0)
T4a	59 (1.0)	42 (1.1)	17 (1.0)
T4b	63 (1.1)	48 (1.2)	15 (0.9)
N			
N0	5505 (97.6)	3845 (97.4)	1660 (98.1)
N1a	74 (1.3)	59 (1.5)	15 (0.9)
N1b	60 (1.0)	43 (1.1)	17 (1.0)
M			
M0	5456 (96.8)	3816 (96.7)	1640 (96.9)
M1	183 (3.2)	131 (3.3)	52 (3.1)

### Establishment of the clinical prediction models

**Fig. 3:**
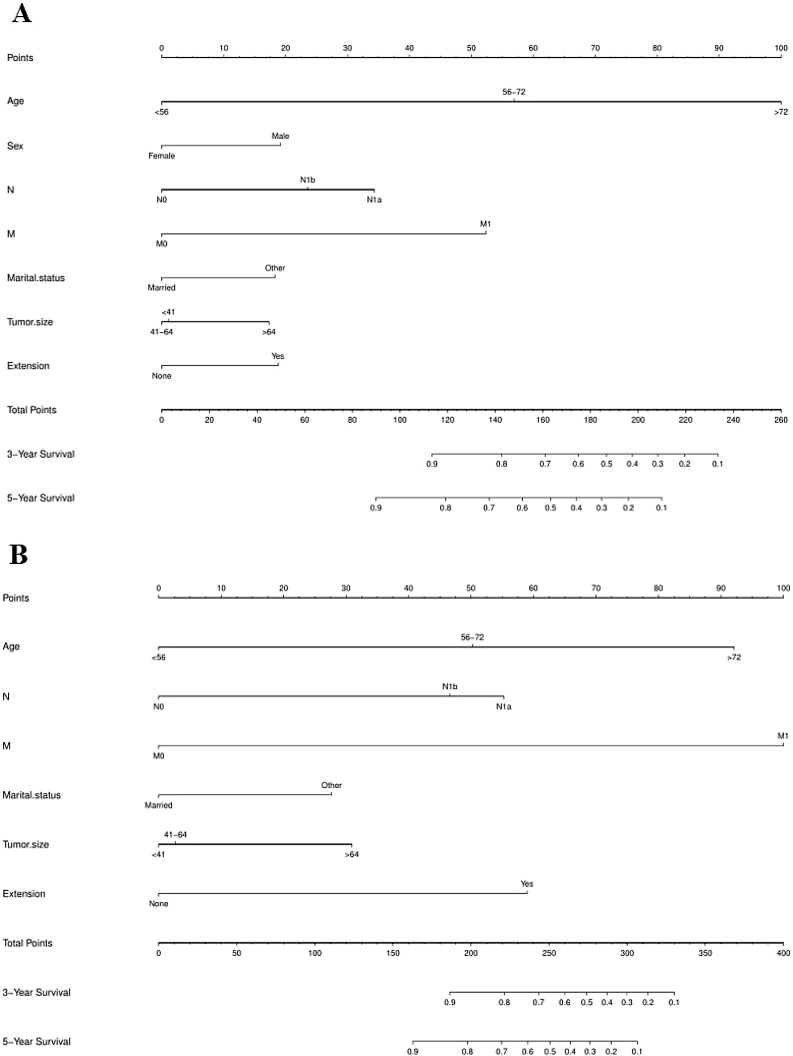
Nomograms to predict 3- and 5-year OS (A) and CSS (B) in postoperative FTC patients.

Univariate analysis of the variables showed that age, sex, marital status, tumor size, gland invasion, number of lesions, and NM stage were significantly correlated with OS. Multivariate analysis of the above 8 variables showed that all the variables except the number of lesions were risk factors affecting FTC prognosis (Table 2). The nomogram of OS was constructed from the above seven variables (Fig. 3A). The principle of the column line diagram is as follows: a perpendicular line is drawn upward to obtain the score of each variable based on the value of each variable of the patient these scores are summed to obtain the total score and then positioned on the axis of the total score. Finally, draw a vertical downward line to identify the 3-year and 5-year OS and CSS, respectively. In univariate analysis, we observed that in addition to sex and the number of lesions, the clinical variables that affect OS also affect CSS, and the results of multivariate analysis of these six variables showed that these six variables were important factors affecting CSS in FTC patients (Table 3). Therefore, the nomogram of CSS was constructed from the above six variables (Fig. 3B).

**Table 2:** Univariate and multivariate analyses of patient OS in the training set.

Characteristics	Univariate analysis		Multivariate analysis	
	HR (95% CI)	p	HR (95% CI)	p
Age				
<56	Reference		Reference	
56–72	5.128 (4.00–6.574)	*<*0.001	4.3005 (3.3403–5.5368)	*<*0.001
>72	17.559 (13.63–22.622)	*<*0.001	13.0450 (10.0468–16.9379)	*<*0.001
Sex				
Male	Reference			
Female	0.5525 (0.4611‒0.6622)	*<*0.001	0.6109 (0.5009‒0.7451)	*<*0.001
Race				
White	Reference			
Black	0.97901(0.7373–1.300)	0.8834		
Other	1.33872 (1.0227–1.752)	0.0337		
Marital status				
Married	Reference			
Unmarried	1.482 (1.239–1.773)	*<*0.001	1.5970 (1.3172–1.9362)	*<*0.001
Tumor size				
<41	Reference			
41‒64	1.437 (1.157–1.784)	0.00102	0.9743 (0.7788–1.2190)	0.819882
>64	3.204 (2.545–4.033)	*<*0.001	1.5291 (1.1922–1.9612)	*<*0.001
Extension				
No	Reference			
Yes	3.398 (2.728–4.233)	*<*0.001	1.5925 (1.2397–2.0456)	*<*0.001
Multifocality				
Unifocal	Reference			
Multifocal	1.412 (1.122–1.775)	0.0032	1.0999 (0.8700–1.3907)	0.426126
Surgery				
Lobectomy	Reference			
Subtotal or near-total thyroidectomy	1.206 (0.8275–1.758)	0.330		
Total thyroidectomy	1.017 (0.8151–1.268)	0.883		
Radiation				
None or refused	Reference			
Radiation Beam or Radioactive implants	3.90980 (2.8028–5.454)	*<*0.001		
Radioisotopes or Radiation beam plus isotopes or implants	0.88990 (0.7374–1.074)	0.224		
T				
T1	Reference			
T2	0.9144 (0.7052–1.186)	0.499406		
T3	1.5558 (1.2131–1.995)	*<*0.001		
T4a	6.4682 (3.9481–10.597)	*<*0.001		
T4b	12.0932 (8.1913–17.854)	*<*0.001		
N				
N0	Reference			
N1a	3.188 (1.963–5.177)	*<*0.001	2.3988 (1.4579–3.9470)	*<*0.001
N1b	6.471 (1.963–5.177)	*<*0.001	1.8116 (1.1575–2.8353)	0.009321
M				
M0	Reference			
M1	9.931 (7.786–12.67)	*<*0.001	3.8438 (2.9313–5.0402)	*<*0.001

CI, Confidence Interval.

**Table 3:** Univariate and multivariate analyses of patient CSS in the training set.

Characteristics	Univariate analysis		Multivariate analysis	
	HR (95% CI)	p	HR (95%CI)	p
Age				
<56	Reference		Reference	
56–72	3.723 (2.407–5.758)	*<*0.001	2.939 (1.8821–4.590)	*<*0.001
>72	13.677 (8.843–21.154)	*<*0.001	7.221 (4.5246–11.524)	*<*0.001
Sex				
Male	Reference			
Female	0.7769 (0.5513–1.095)	0.149		
Race				
White	Reference			
Black	1.371 (0.8572–2.193)	0.1879		
Other	1.708 (1.0681–2.733)	0.0254		
Marital status				
Married	Reference			
Unmarried	1.978 (1.426–2.744)	*<*0.001	1.811 (1.2977–2.528)	*<*0.001
Tumor size				
<41	Reference			
41‒64	1.653 (1.091–2.507)	0.0178	1.060 (0.6908–1.626)	0.790110
>64	5.589 (3.824–8.167)	*<*0.001	1.942 (1.2762–2.956)	0.001948
Extension				
No	Reference			
Yes	8.487 (6.087–11.84)	*<*0.001	3.545 (2.4373–5.156)	*<*0.001
Multifocality				
Unifocal	Reference			
Multifocal	1.453 (0.9502–2.222)	0.0847		
Surgery				
Lobectomy	Reference			
Subtotal or near-total thyroidectomy	1.747 (0.8369–3.646)	0.1374		
Total thyroidectomy	1.594 (0.9909–2.565)	0.0545		
Radiation				
None or refused	Reference			
Radiation Beam or Radioactive implants	12.847 (8.0579–20.48)	*<*0.001		
Radioisotopes or Radiation beam plus isotopes or implants	1.212 (0.8298–1.77)	0.32		
T				
T1	Reference			
T2	1.380 (0.7384–2.578)	0.313		
T3	3.617 (2.0314–6.439)	*<*0.001		
T4a	27.414 (12.8792–58.353)	*<*0.001		
T4b	48.948 (24.2960–98.614)	*<*0.001		
N				
N0	Reference			
N1a	6.791 (3.561–12.95)	*<*0.001	3.272 (1.6871–6.345)	*<*0.001
N1b	14.877 (8.547–25.90)	*<*0.001	2.721 (1.4894–4.970)	0.001131
M				
M0	Reference			
M1	24.82 (17.58–35.03)	*<*0.001	8.556 (5.8277–12.561)	*<*0.001

CI, Confidence Interval.

### Validation of clinical predictive models

**Fig. 4:**
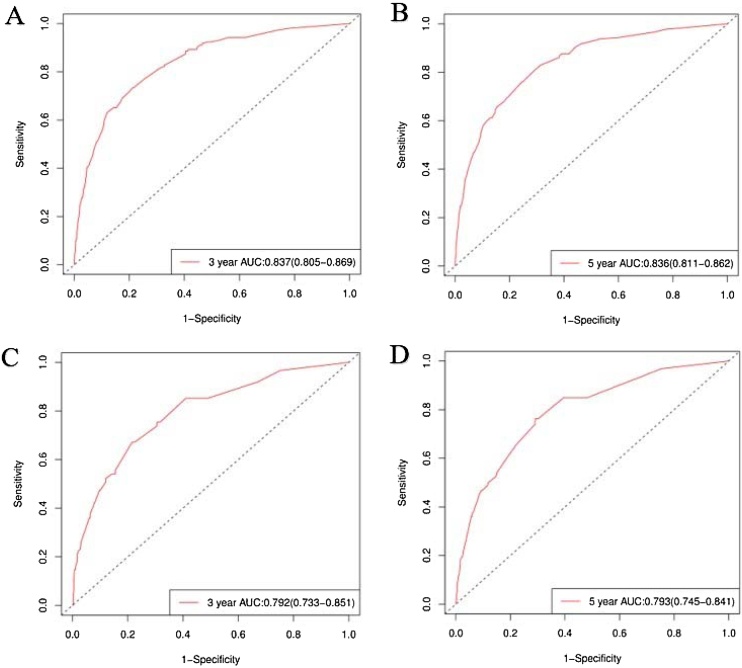
ROCs was used to assess 3- and 5-year OS in the training (A‒B) and validation (C‒D) groups.

**Fig. 5:**
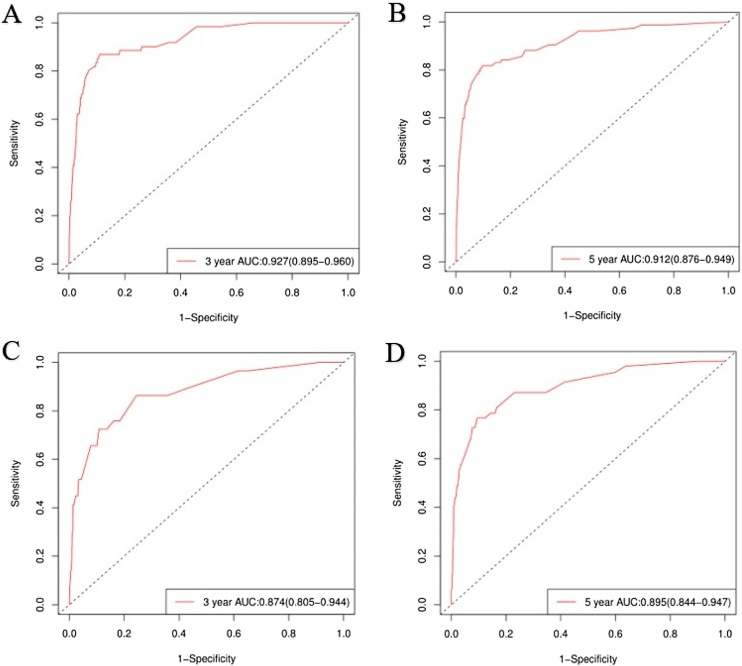
ROCs was used to assess 3- and 5-year CSS in the training (A‒B) and validation (C‒D) groups.

**Fig. 6:**
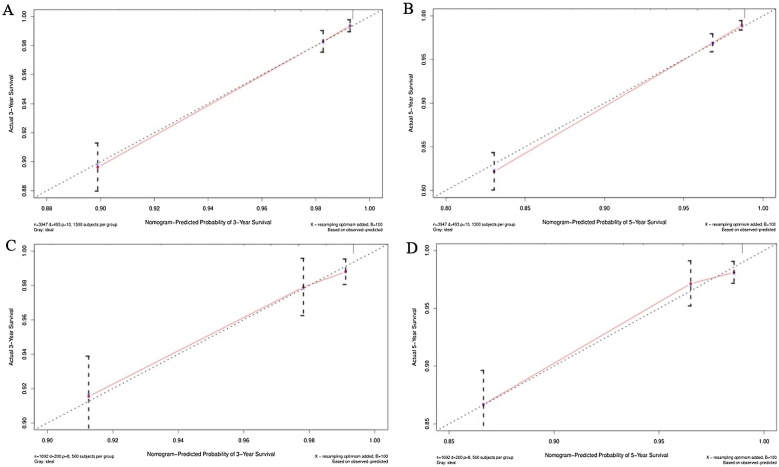
Calibration plots was used to assess 3- and 5-year OS in the training (A‒B) and validation (C‒D) groups.

**Fig. 7:**
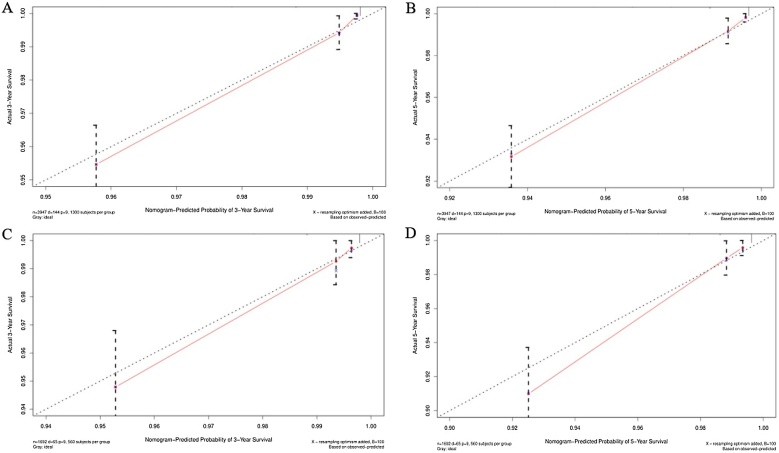
Calibration plots was used to assess 3- and 5-year CSS in the training (A‒B) and validation (C‒D) groups.

The accuracy of the column line graphs was assessed using the C-index, which was 0.821 (95% CI 0.801‒0.840) for OS and 0.888 (95% CI 0.859‒0.917) for CSS of the prediction model in the training set, while the C-indexes for OS and CSS of the prediction model in the validation set were 0.791 (95% CI 0.756‒0.826) and 0.895 (95% CI 0.853‒0.936), respectively. Next, the sensitivity of the column line graph was verified by ROC curves, and the two ROC models for 3-year and 5-year OS were compared in the training and validation sets, respectively, and the results showed that the AUCs for 3-year and 5-year OS in the training set were 0.837 and 0.836, respectively, and the AUCs for 3-year and 5-year OS in the validation set were 0.792 and 0.793, respectively (Fig. 4). The two ROC models for 3-year and 5-year CSS were then compared in the training set, and the results showed that the AUCs of 3-year and 5-year CSS predicted by the column-line graph prediction model were 0.927 and 0.912 in the column-line graph prediction model, while the AUCs of 3-year and 5-year CSS were 0.874 and 0.895 in the validation set (Fig. 5). The calibration curves were used to verify the accuracy of the nomograms for OS (Fig. 6) and CSS (Fig. 7). This is judged by comparing the overlap between the prediction curve and the standard curve in the calibration plot; the higher the overlap between the two, the better the calibration of the prediction model. Through the dual validation of ROCs and calibration plots, the results illustrate that the column-line diagrams established in this study are highly accurate and can predict OS and CSS for FTC patients with different conditions.

## Discussion

Currently, there are differences in the results of domestic and international studies on the prognostic factors for the survival of FTC patients. Zhang et al.^13^ found significant risk factors associated with death in patients with FTC: age, sex, multifocality, tumor size, extra-thyroidal extension, extensive infiltration, metastasis and surgical status. Teo et al.^14^ reported that age, tumor size, vascular infiltration, tumor invasiveness, distant metastasis, resection completeness, and external radiation were important factors for the survival of FTC patients. In contrast, Wang et al.^15^ found no significant effect of gender and race on the risk of FTC death. In our study, age, sex, marital status, tumor size, glandular invasion, lymph nodes, and distant metastasis were important factors affecting OS, and the above six variables in addition to sex were also important factors affecting CSS. Age was categorized as <56-years, 56–72 years and >72-years by X-tile software, and multifactorial Cox proportional risk regression analysis revealed that in terms of overall survival, FTC patients aged 56–72 years (HR = 4.3005, 95% CI 3.3403–5.5368), >72-years (HR = 13.0450, 95% CI 10.0468–16.9379), and cancer-specific survival FTC patients were aged 56–72 years (HR = 2.939, 95% CI 1.8821–4.590), >72 years (HR = 7.221, 95% CI: 4.5246–11.524). Many reports have showed that age at diagnosis is a key factor in the survival of patients with FTC, with the odds of death generally increasing with age.^13^^,^^16^

The incidence of thyroid cancer is greater in women than in men, with an incidence more than twice as high as that in men.^17^ However, the prognosis of women with thyroid cancer in women is usually more favorable than that of in men with thyroid cancer.^18^ In the present, there were 2.3 times female FTC patients than male FTC patients. Previous researches have showed that sex steroid hormones may influence the development of thyroid cancer in women. Schonfeld et al.^19^ explored the association between polymorphic variants in hormonal pathway genes and the development of PTC and found no strong evidence of a close association between polymorphic variants in hormonal pathway genes and PTC. A lack of consistency in the correlation between hormonal and reproductive factors and PTC was also shown in another study.^20^^,^^21^ Therefore, the high female prevalence of thyroid cancer remains a mystery.

Several studies have reported that marital status have an important impact on survival in oncology patients, and our findings also demonstrate that marital status is a key factor influencing FTC. Ai et al.^22^ explored the impact of marital status on the prognosis of patients with medullary thyroid carcinoma and showed that unmarried patients had a greater risk of death than married patients and that marriage had an important protective effect on patients older than 52-years of age, which diminished with the progression of the tumor. Shi et al.^23^ investigated the effect of marriage on DTC outcomes and showed that being unmarried, especially being widowed, increased the odds of dying from DTC. An exploration of the associations between marital status and prognosis in patients with other cancers, including breast, lung, colorectal, esophageal, and gastric cancers revealed similar findings.^24^, ^25^, ^26^, ^27^ Studies have reported a direct correlation between tumor size and poor prognosis. Ywata et al.^28^ showed a strong correlation between tumor diameter >10 mm and tumor recurrence. Similarly, Zhang et al.^14^ found a higher incidence of death in FTC patients with tumors >4 cm in diameter than those ≤4 cm in diameter. In this research, tumor size was classified as <41 mm, 41–64 mm, and >64 mm by X-tile software, where the survival rate was lower for tumors >64 mm, which had similar results to previous studies. On the basis of the 2017 WHO guidelines, the degree of FTC invasion was classified as microinfiltration (miFTC), encapsulated angioinvasive with perivascular infiltration of FTC (eaFTC), or extensive infiltration (wiFTC).^29^ With minimal infiltration, the tumor infiltrates only the peritoneum; with peritoneal vascular infiltration, the tumor is accompanied by vascular infiltration (with or without peritoneal infiltration); and with extensive infiltration, the tumor infiltrates extensively or locally via the capsule into the thyroid and extrathyroidal tissues. D'Avanzo et al.^30^ studied the clinical course of 132 patients with FTC to determine whether there was a direct relationship between the degree of histological infiltration, tumor recurrence, and patient survival. The authors demonstrated that patients with extensively infiltrated FTC had the highest overall mortality. In a retrospective study of FTC in children and adolescents, peritumoral infiltration and vascular infiltration were found to have an important impact on the outcome of patients with FTC.^31^ A recent study revealed that the tumor envelope influences the prognosis of patients with low DTC and that enveloped tumors without envelope infiltration have a better prognosis in terms of disease progression.^32^ Tumor infiltration was shown to be a correlate of OS and CSS prognosis in FTC patients in this study.

Whether lymph node metastasis affects the progression of FTC remains questionable.^33^^,^^34^ Witte et al.^35^ investigated whether lymph node metastasis affects the prognosis of FTC, and the results showed that it had an important impact on disease recurrence and patient survival. In this study, we found that most patients did not have lymph node metastasis, but lymph node metastasis occurred, it had an important impact on the prognosis of all FTC patients. According to previous studies, distant metastasis has been proven to be a significant factor in the prognosis of FTC patients.^36^ FTC usually invades blood vessels through the bloodstream to the bones and lungs. In a retrospective study, Su et al.^37^ collected data on 204 patients with FTC diagnosed in Taiwan, and a multifactorial analysis revealed that distant metastasis was the most important factor in the risk of death and cumulative incidence of FTC. Sampson et al.^38^ evaluated 49 DTC patients with distant metastases and reported that survival was significantly affected by the site of distant metastases, with lung metastases usually having a better prognosis than bone metastases. In our study, distant metastasis had a critical impact on the survival of patients with FTC.

We constructed column-line plots based on the above seven variables. The strength of this study was the inclusion of many patients, which allowed screening of eligible patients. More predictors were evaluated by unifactorial and multifactorial analyses, which led to the selection of meaningful predictors to build models with clinical applications. The variables of our column-line diagrams were readily available in the clinical data. The column-line plots performed well in predicting patients' postoperative OS and CSS and were validated by ROC curves and calibration curves. The nomogram, a statistical visualization tool integrating multiple predictive indicators, is highly valuable for predicting tumor prognosis. It facilitates individualized risk assessment and provides precise support for clinical decision-making. However, our research has its drawbacks. First, there is a certain bias due to the unavailability of certain information about the patients in the SEER database, even because incomplete recording of clinical data of some patients will lead to the deletion of the data, and there is a bias between the data collected and the original clinical data in the end. Second, the data we extracted for the study did not include variables such as serum calcitonin, Carcinoembryonic Antigen (CEA), BRAF point mutation or TERT promoter point mutations, which cannot predict the role of these mutations in FTC. Although it has inevitable drawbacks, it provides important reference value for the disease development of cancer patients.

## Conclusion

We successfully established 3- and 5-year OS and CSS in postoperative FTC patients with a nomogram, which helps to evaluate the prognosis of the disease and subsequent treatment options.

## ORCID IDs

Xin Liu: 0009-0007-3343-6352

Suidan Chen: 0000-0002-5226-8573

Cangui Wu: 0009-0002-0755-7664

## Declaration of competing interest

The authors declare no conflicts of interest.
